# Nascent Extracellular Matrix Converts Biomaterial Cues into Cell Fate Decisions

**DOI:** 10.1002/advs.77895

**Published:** 2026-09-27

**Authors:** Joshua Y. Liu, Ziqian Yang, Arina E. Velieva, Eleanor M. Plaster, Mingyue Fan, Donia W. Ahmed, Avinava Roy, Pamela Duran, Pamela Panovich, Alexandra S. Piotrowski‐Daspit, Carlos A. Aguilar, Megan L. Killian, Claudia Loebel

**Affiliations:** ^1^ Department of Bioengineering University of Pennsylvania Philadelphia USA; ^2^ Center for Precision Engineering for Health (CPE4H) University of Pennsylvania Philadelphia USA; ^3^ Department of Biomedical Engineering University of Michigan Ann Arbor USA; ^4^ Department of Materials Science & Engineering University of Michigan Ann Arbor USA; ^5^ Internal Medicine ‐ Pulmonary & Critical Care Medicine Division University of Michigan Ann Arbor USA; ^6^ Department of Orthopaedic Surgery University of Michigan Ann Arbor USA

**Keywords:** biomaterial, cell biology, cell fate determination, cell growth, cellular differentiation, chemistry, extracellular matrix

## Abstract

Hydrogels serve as powerful models for investigating cell‐extracellular matrix (ECM) interactions. While chemical modifications are routinely used to tune hydrogel properties, it remains unclear whether these modifications mediate cell fate. Previous work has shown that cells deposit newly synthesized (nascent) ECM at the cell‐hydrogel interface. Here, we demonstrate that this nascent ECM interface regulates how cells interpret chemical modifications. Using hydrogels with varied chemical modifications, we isolated the effects of chemical modification on nascent ECM and cell fate. Nascent ECM deposition increased as a function of hydrogel modification and with distinct matrisome compositions. While low‐modification hydrogels promoted cell differentiation, high modifications increased cell proliferation. Perturbing cell‐ nascent ECM interactions reversed this cell fate. Our findings reveal that nascent ECM regulates cell fate by converting hydrogel cues into signals that control cell fate. This tri‐directional interplay among hydrogel chemical modifications, nascent ECM, and cell fate reframes how we interpret cell‐hydrogel interactions.

## Graphical abstract

This study uncovers a tri‐directional interplay among cells, hydrogels, and their nascent extracellular matrix. Hydrogel norbornene modification alters CD44‐mediated cell–hydrogel interactions, which shape nascent matrix deposition, composition, and spatial organization. In turn, β1‐integrin‐dependent engagement with this evolving nascent matrix microenvironment regulates chondrocyte proliferation and differentiation. These findings begin to reveal how biomaterial cues are converted into cell‐fate decisions.

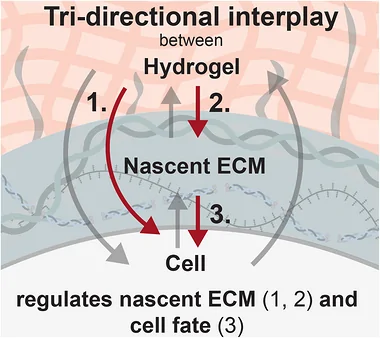

## 1 Introduction

The extracellular matrix (ECM), an organized assembly of structural and signaling molecules, integrates mechanical and biochemical signals to regulate cellular responses in tissue homeostasis and disease progression [1]. Engineered hydrogels have emerged as powerful tools to recapitulate key features of the ECM, including the mechanical and biochemical signals that cells are exposed to. Building upon the concept of bi‐directional cell‐ECM signaling (Mina Bissel, 1982 [2, 3]), these hydrogels have been instrumental in investigating how the ECM instructs cell function and how in turn cells remodel the ECM. However, recent work has challenged this bi‐directional framework by demonstrating that cells, upon embedding into hydrogels, rapidly deposit a layer of newly synthesized (nascent) ECM (nECM) at the cell‐hydrogel interface [4, 5, 6, 7]. Importantly, cellular interactions with this nECM have been shown to regulate mechanosensing [8, 9] that directs downstream cell function such as cell spreading and differentiation [4, 10]. Moreover, nECM accumulation has been reported to support the formation and growth of three‐dimensional multicellular structures including spheroids and organoids in vitro [10, 11]. While hydrogel mechanical properties are known to regulate the thickness of deposited nECM [8, 10, 12], how the hydrogel polymer backbone itself contributes to nECM deposition and cell function remains unexplored. Thus, there is a critical gap in our fundamental knowledge on the role of hydrogel‐nECM interactions in regulating cell function and fate.

The fabrication of hydrogels often requires chemical modifications of the polymer backbone (e.g., methacrylates/acrylates, norbornenes, or vinyl‐sulfones) to form crosslinks and provide handles for biochemical signals (e.g., RGD (Arginine‐Glycine‐Aspartic acid), peptides or full‐length proteins) [13, 14]. Importantly, these modifications may also alter hydrogel properties beyond crosslinking, including polymer hydrophobicity, protein adsorption, swelling, and diffusivity, which may in turn influence cell behavior and matrix organization [15, 16, 17, 18, 19]. While recent work highlighted that RGD ligand presentation reduces nECM deposition by spheroids in methacrylated hyaluronic acid hydrogels [6], whether the methacrylates themselves alter nECM deposition remains unknown. Interestingly, a previous study suggested that increasing the degree of modification of hyaluronic acid hydrogels impairs chondrogenic differentiation of mesenchymal stromal cells. This was attributed to reduced CD44‐hyaluronic acid engagement resulting from functional group substitution [20]. Yet, how the chemical modifications of the hydrogel polymer backbone alter nECM deposition, composition, and its functional properties are unknown.

To address this knowledge gap, we synthesized hyaluronic acid polymers with varying degree of norbornene modification to fabricate hydrogels with matched crosslinker concentration and mechanical properties. Building upon previous studies [9, 21, 22], we used metabolic labeling to characterize the spatiotemporal evolution and the structure and composition of cell‐deposited nECM as a function of chemical modifications. In addition, we performed perturbation studies to dissect the specific contributions of cell‐hydrogel and cell‐nECM interactions in guiding cell fate decisions. Our findings show that hydrogel norbornene modification, independent of mechanical properties, regulated nECM deposition and cell fate. This work challenges the traditional bi‐directional interaction between cells and engineered hydrogels and instead provides evidence for a tri‐directional interplay among hydrogels, nECM, and cells within 3D hydrogel culture.

## 2 Results and Discussion

### 2.1 Hydrogel Modifications Direct nECM Deposition

**FIGURE 1:**
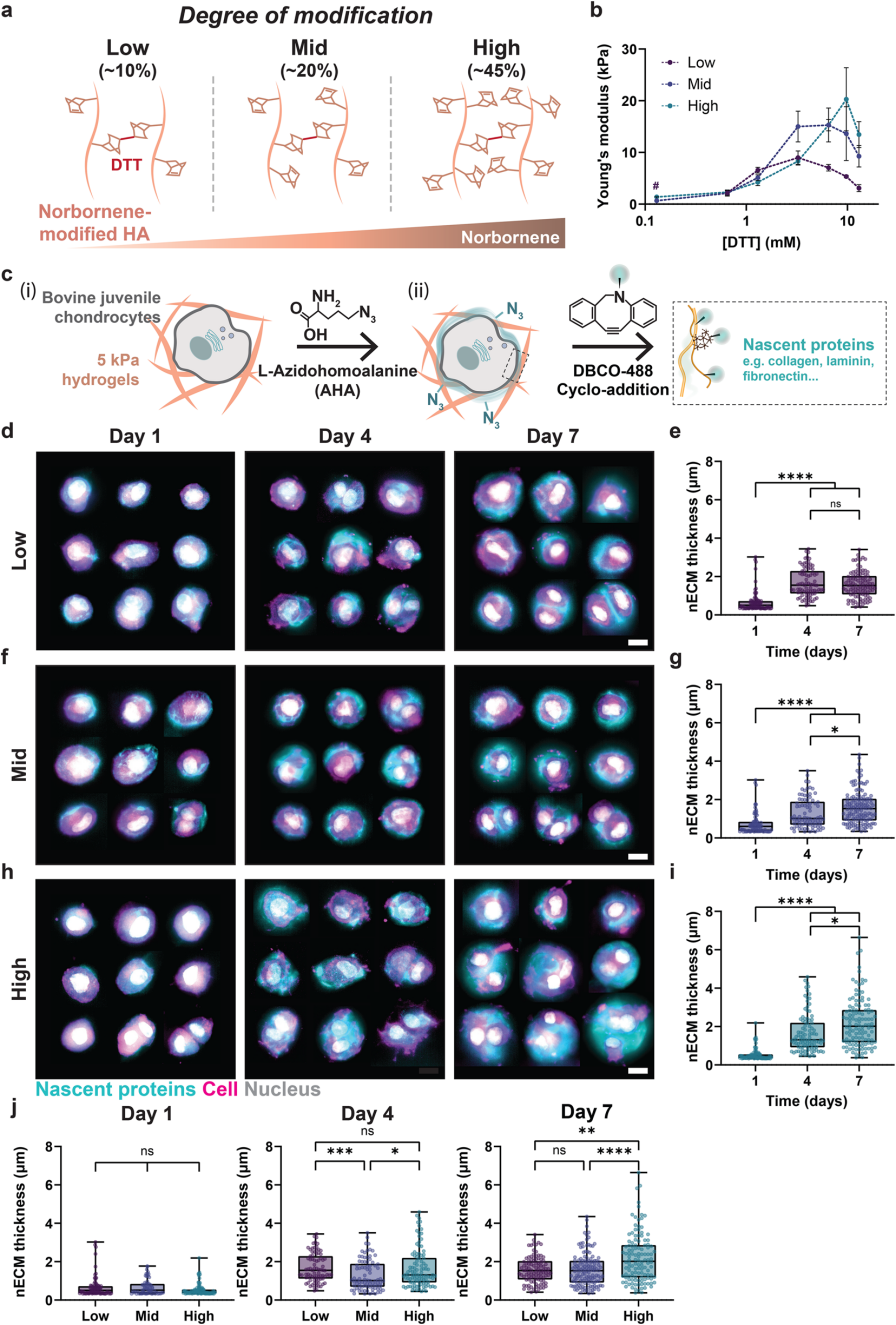
Hydrogel modifications direct nECM deposition and accumulation. (a) Schematic illustrating the design of hydrogels with varying degrees of norbornene chemical modification (low, mid, high). (b) Compressive modulus of hydrogels as a function of crosslinker concentration, measured via unconfined compression testing (*n* = 3–4 hydrogels per group, *N* = 3, Day 1). ^#^ Not measurable due to lack of structural integrity (low, 0.13 µM). (c) Schematic of NorHA hydrogel embedded bovine juvenile chondrocytes at 5×10^6^/mL seeding density (i), cultured in media supplemented with azidohomoalanine (AHA), prior to fluorophore‐conjugated DBCO click chemistry to visualize nascent extracellular matrix (nECM, iii): (d) Representative fluorescent images and (e) quantification of average nECM thickness of chondrocytes cultured in low‐modification hydrogels at day 1, day 4, and day 7 (scale bar = 10 µm, day 1 *n* = 85 cells, *N* = 2; day 4 *n* = 82 cells, *N* = 2; day 7 *n* = 101 cells, *N* = 3). (f) Representative fluorescent images and (g) quantification of average nECM thickness of chondrocytes cultured in mid‐modification hydrogels at day 1, day 4, and day 7 (scale bar = 10 µm, day 1 *n* = 86 cells, *N* = 2; day 4 *n* = 86 cells, *N* = 2; day 7 *n* = 136 cells, *N* = 3). (h) Representative fluorescent images and (i) quantification of average nECM thickness of chondrocytes cultured in high‐modification hydrogels at day 1, day 4, and day 7 (scale bar = 10 µm, day 1 *n* = 89 cells, N = 2; day 4 *n* = 102 cells, *N* = 2; day 7 *n* = 122 cells, *N* = 3). (j) Quantification of average nECM thickness of chondrocytes cultured in low, mid and high modification hydrogels at day 1, 4, 7 (Day 1: low *n* = 85, *N* = 2; mid *n* = 86, *N* = 2; high *n* = 89, *N* = 2; Day 4: low *n* = 82, *N* = 2; mid n = 86, *N* = 2; high *n* = 102, *N* = 2; Day 7: low *n* = 101, *N* = 3; mid *n* = 136, *N* = 3; high *n* = 122, *N* = 3) (a–j) *N* = number of independent experiments, error bar in mean line plots = standard deviation, ^****^ *p* < 0.0001, ^***^ *p* < 0.001, ^**^ *p* < 0.01, ^*^ *p* < 0.05, ns: not significant by the Kruskal–Wallis test with Dunn's multiple comparisons test, box plots = median, interquartile range, and minimum–maximum values.

To probe how hydrogel modifications direct nECM deposition, we engineered norbornene‐modified hyaluronic acid (NorHA) hydrogels with ‘low’ (∽10%), ‘mid’ (∽23%), and ‘high’ (∽43%) degree of norbornene modification (Figure 1a). NorHA hydrogels were then crosslinked via thiol‐ene reaction using dithiothreitol (DTT), and the mechanical properties were measured via compression testing. Using 2 wt.% NorHA, increasing the DTT concentration increased the Young's modulus across groups (Figure 1b). However, the Young's moduli saturated at different DTT concentrations depending on the degree of norbornene modification. For ‘low’ modification hydrogels, saturation occurred at approximately 3.25 mM DTT, whereas ‘mid’ modification saturated at 6.5 mM DTT and ‘high’ modification at 9.75 mM DTT. This is expected due to the differences in the amount of norbornene available for crosslinking [21]. In contrast, hydrogels crosslinked with 0.65 and 1.3 mM DTT exhibited comparable Young's moduli. Thus, to investigate the contributions of hydrogel modification independent of mechanical properties, we selected 1.3 mM DTT to obtain 5 kPa NorHA hydrogels with ‘low’, ‘mid’, and ‘high’ norbornene modification. Next, we embedded juvenile bovine chondrocytes in 5 kPa ‘low’, ‘mid’ and ‘high’ NorHA hydrogels and cultured the constructs in methionine‐free, L‐azidohomoalanine (AHA) containing media, which enabled the visualization and analysis of newly synthesized proteins using copper‐free strain‐promoted azide‐alkyne cycloaddition with a DBCO (Dibenzocyclooctyne)‐fluorophore [9] (Figure 1c). Within one day of culture, DBCO staining showed a thin and discontinuous nECM layer around cells within all NorHA hydrogels, which then continuously increased in thickness over 7 days of culture (Figure 1d). Within ‘low’ hydrogels, quantification of nECM thickness showed an almost twofold increase from 0.68 ± 0.54 µm (Day 1) to 1.68 ± 0.74 µm at day 4 but no further increase till day 7 (1.58 ± 0.67 µm, Figure 1e), indicating that nECM deposition plateaued within the first few days of culture. In contrast, nECM thickness of cells cultured within ‘mid’ NorHA hydrogels continuously increased from 0.74 ± 0.55 µm (day 1) to 1.30 ± 0.78 µm (day 4), and 1.59 ± 0.85 µm (day 7), suggesting prolonged nECM deposition when compared to ‘low’ hydrogels (Figure 1f). In ‘high’ hydrogels, nECM thickness was initially comparable to ‘low’ and ‘mid’ hydrogels at Day 1 (0.54 ± 0.31µm) but showed even more significant increases between the time points (Figure 1g–i, day 4: 1.66 ± 1.01 µm, day 7: 2.21 ± 1.26 µm). When comparing nECM thickness across groups, at day 7 we observed a 1.5‐fold increase in nECM thickness between cells cultured in the ‘high’ hydrogels when compared to both ‘low’ and ‘mid’ hydrogels, with little difference at day 1 and day 4 (Figure 1j). Notably, assessing nECM thickness at the single‐cell level showed little heterogeneity at day 1, whereas by day 7, cells in ‘high’ hydrogels showed a strong increase in heterogeneity as well Figure S1a–c). These data show that hydrogel modifications further regulate the heterogeneity of nECM accumulation.

Taken together, these findings demonstrate that hydrogel modifications guide nECM deposition and heterogeneity throughout the culture time. More specifically, ‘low’ hydrogels promote faster, but overall lower nECM deposition compared to ‘high’ hydrogels with sustained and increased nECM accumulation over time.

### 2.2 Hydrogel Modifications Regulate Cell Proliferation, Morphology, and Differentiation

**FIGURE 2:**
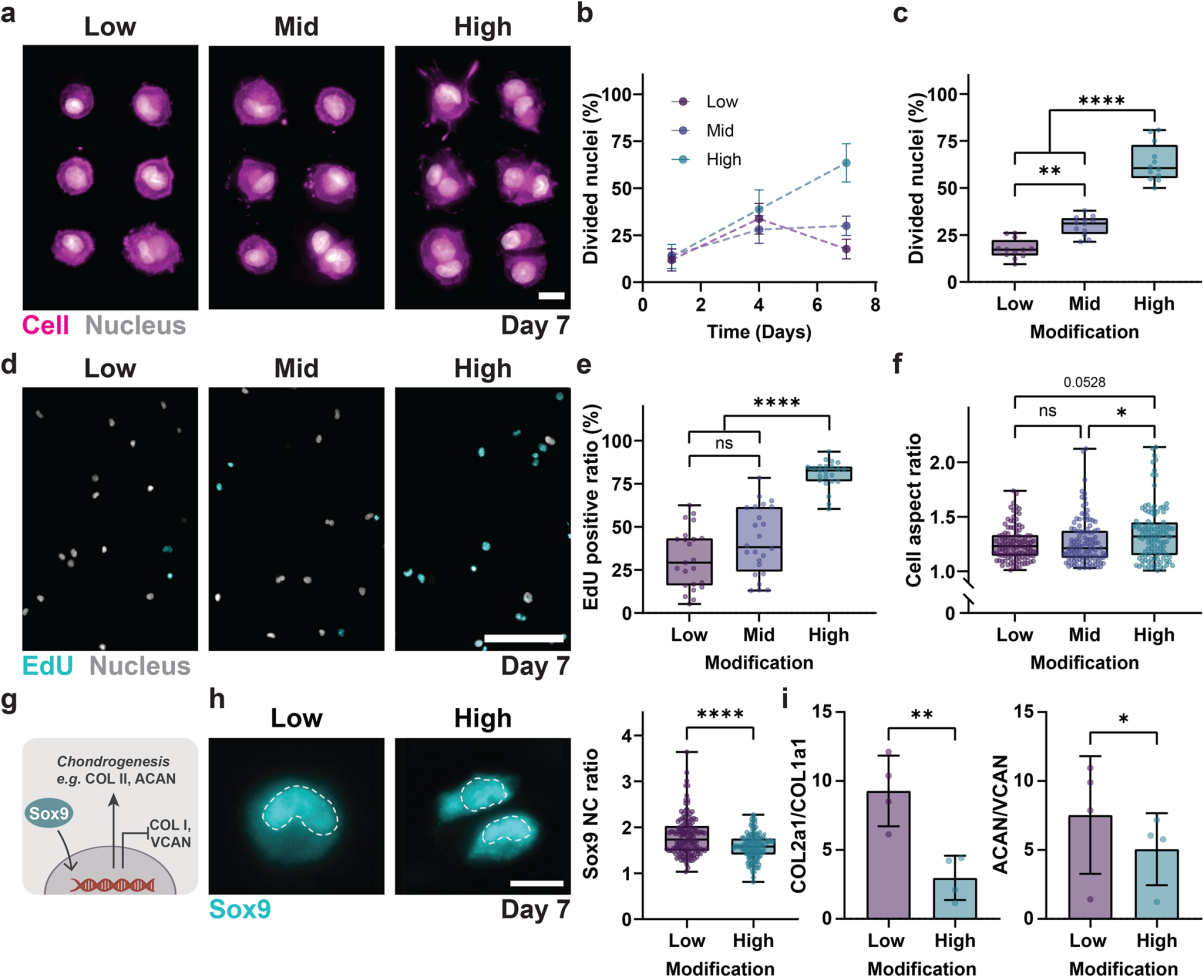
Hydrogel modifications regulate cell fate. (a) Representative fluorescent images of nuclei and cell membrane of chondrocytes cultured in low, mid, and high modification hydrogels at day 7 (scale bars: 10 µm.) (b) Quantification of divided nuclei of chondrocytes culture in low, mid and high modification hydrogels at day1, 4 and 7 (low: day 1 *n* = 12 regions of interest (ROIs), *N* = 2; day 4 *n* = 9 ROIs, *N* = 2; day 7 *n* = 12 ROIs, *N* = 3; mid: day 1 *n* = 8 ROIs, *N* = 2; day 4 *n* = 8 ROIs, *N* = 2; day 7 *n* = 12 ROIs, *N* = 3; high: day 1 *n* = 10 ROIs, *N* = 2; day 4 *n* = 9 ROIs, *N* = 2; day 7 *n* = 12 ROIs, *N* = 3) (c) Quantification of divided nuclei of chondrocytes culture in low, mid and high modification hydrogels at day 7 (low: *n* = 12 ROIs, *N* = 3; mid: *n* = 12 ROIs, *N* = 3; and high: *n* = 12 ROIs, *N* = 3). (d) Representative fluorescent images of incorporation of 5‐ethynyl‐2´‐deoxyuridine (EdU) into chondrocytes cultured in low, mid, and high modification hydrogels at day 7 (scale bar: 100 µm). (e) Quantification of EdU incorporation into chondrocytes cultured in low, mid and high‐modification hydrogels at day 7 (scale bar: 100 µm, low: *n* = 23 ROIs, *N* = 4; mid: *n* = 24 ROIs, *N* = 4; and high: *n* = 23 ROIs, *N* = 4). (f) Quantification of chondrocyte cell aspect ratio at day 7 (low: *n* = 108 cells, *N* = 3; mid: *n* = 112 cells, *N* = 3; and high: *n* = 111 cells, *N* = 3). (g) Schematic showing the downstream effects of Sox9 translocation into the nucleus, including upregulation of collagen type IIA1, aggrecan (ACAN) and Sox9, and the downregulation of collagen type IA1, and versican (VCAN) (h) Representative immunofluorescent images and quantification of Sox9 nuclear translocation calculated by the nucleus‐to‐cytoplasm (NC) ratio at day 7 (scale bar: 10 µm, low: *n* = 139 cells, *N* = 3; and high: *n* = 124 cells, *N* = 3). (i) Ratio of COL2A1/COL1A1 and ACAN/VCAN gene expression at day 7, measured by qPCR and normalized to the S18 housekeeping gene. Gene expression ratios were calculated as 2^(‐(ΔCt^_1_^– ΔCt^_2_^))^, where ΔCt_1_ and ΔCt_2_ represent Ct values of each gene normalized to S18. (*N* = 4) (a–i) *N* = number of independent experiments, error bar in bar plots = standard deviation, box plots = median, interquartile range, and minimum–maximum values, ^****^ *p* < 0.0001, ^**^ *p* < 0.01, ^*^ *p* < 0.05, ns: not significant. c, one‐way ANOVA with Tukey's multiple comparisons test; e–f, Kruskal–Wallis test with Dunn's multiple comparisons test; h, two‐tailed Mann–Whitney test; i, two‐tailed paired Student's *t* test.

After having shown that hydrogel modifications direct the amount and heterogeneity of deposited nECM, we next sought to investigate cellular responses. Hoechst staining at day 7 revealed a higher number of cells with divided nuclei in ‘high’ hydrogels when compared to cells cultured in ‘low’ and ‘mid’ hydrogels (Figure 2a). Quantification of the percentage of divided cell nuclei per total number of cells as a function of hydrogel modification showed a continuous increase over the 7 days of culture, reaching up to 63% ± 10% of cells with divided nuclei in ‘high’ hydrogels (Figure 2b). In contrast, cells in the ‘low’ and ‘mid’ hydrogels showed an initial increase in dividing nuclei, reaching 34% ± 8% (low) and 28% ± 7% (mid) at day 4. After this point, no further increase was observed. When comparing the number of divided nuclei per cell at day 7, we measured an almost threefold increase between ‘low’ and ‘high’ hydrogels (Figure 2c), suggesting that hydrogel modifications have a direct effect on cell division. Increased cell proliferation was further confirmed by 5‐ethynyl‐2´‐deoxyuridine (EdU) incorporation over 7 days of culture (Figure 2d). While we observed proliferating cells in all hydrogels, the percentage of EdU‐positive cells showed up to a 2.5‐fold increase in ‘high’ hydrogels (80% ± 8%) when compared to ‘low’ hydrogels (32% ± 17%, Figure 2e). No differences were observed in nuclear localization of Yes‐associated protein (YAP) between ‘low’ and ‘high’ hydrogels (Figure S2). These results suggest that an increase in hydrogel modifications increases cell proliferation independent of YAP. Interestingly, the higher proliferative capacity correlated with higher cell aspect ratios on day 7 (Figure 2f), suggesting that hydrogel modifications may also direct cell elongation during proliferation.

We next sought to determine whether hydrogel modifications alter downstream chondrogenic differentiation, focusing on transcription factor Sox9 (SRY‐Box Transcription Factor 9), its downstream transcriptional targets *COL2A1* (Collagen type II), *ACAN* (Aggrecan), and indirect suppression of *COL1A1* (Collagen type I), *VCAN* (Versican) in ‘low’ and ‘high’ hydrogels [23, 24] (Figure 2g). At day 7, immunofluorescence showed increased Sox9 protein nuclear staining and an increase in Sox9 nuclear‐to‐cytoplasmic ratios for cells cultured in ‘low’ hydrogels compared to ‘high’ hydrogels (Figure 2h). Gene expression of Sox9 was similarly increased in ‘low’ hydrogels compared to ‘high’ hydrogels (Figure S3). Cells in ‘low’ hydrogels showed a 3.5‐fold increase in COL2A1/COL1A1 and a 1.4‐fold increase in the ACAN/VCAN ratios when compared to ‘high’ hydrogels (Figure 2i), reflecting enhanced chondrogenic differentiation [25]. These results demonstrate that ‘low’ hydrogels show a higher tendency to maintain chondrogenic phenotype, compared to ‘high’ hydrogels that promote cell proliferation.

Several previous studies have investigated how hydrogel properties contribute to chondrogenesis and matrix deposition. For example, faster stress relaxation has been suggested to promote chondrogenic differentiation by reducing mechanical confinement [12]. It has also been reported that the hydrophobicity of chemical groups such as norbornenes may alter deposited protein adsorption [18, 26, 27], and hydrogel swelling can enhance ECM distribution by facilitating molecular diffusion [19]. However, hydrogels with ‘low’ and ‘high’ norbornene modifications showed similar stress relaxation, protein adsorption, and diffusivity, with only a small reduction in equilibrium swelling in the ‘high’ hydrogels compared with the ‘low’ hydrogels (Figure S4). These data indicate that the degree of norbornene modification has only a minor effect on the measured bulk hydrogel properties. Note that swelling reached an equilibrium within 24 h, whereas differences in nECM deposition developed over several days. Together with the opposing relationship between swelling and nECM accumulation, these findings suggest that swelling is less likely to be the primary contributor to the differences in matrix deposition. Importantly, chondrocytes cultured in hydrogels with higher elastic modulus (10 kPa) similarly showed increased chondrogenic differentiation with reduced nECM deposition in ‘low’ when compared to ‘high’ hydrogels (Figure S5). These findings established that the degree of norbornene modifications is linked with differences in proliferative and chondrogenic cell states.

### 2.3 Initial Hydrogel Cues Preserve Cell Phenotype

**FIGURE 3:**
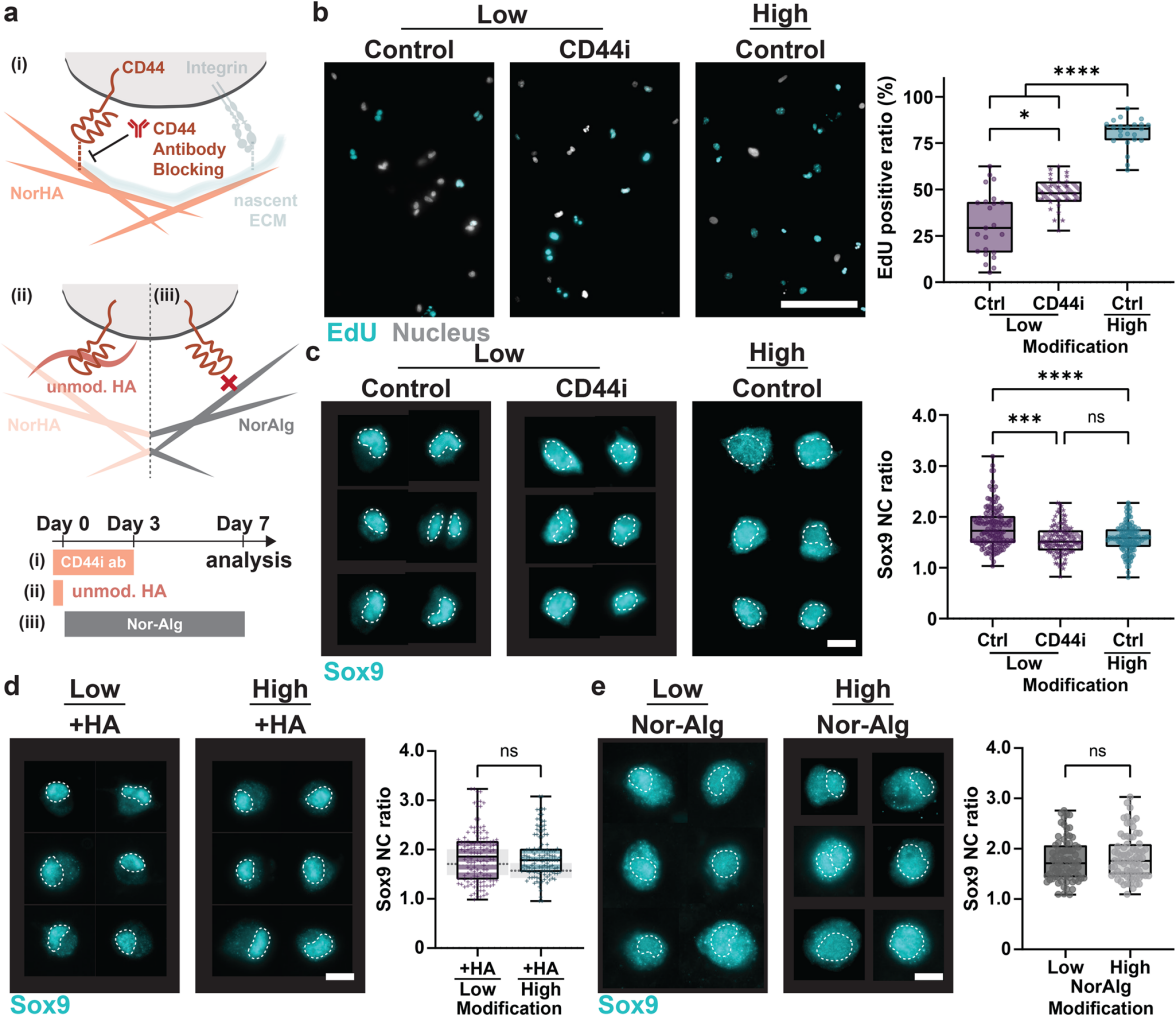
Cellular interactions with the hydrogel backbone are required for maintaining cell fate. (a) Timeline and schematic illustrating three approaches to perturb CD44–hydrogel interactions: (i) CD44 blocking from 1 h pre‐embedding through day 3, (ii) unmodified HA pre‐incubation before embedding, and (iii) culture in NorAlg hydrogels through day 7. (b) Representative fluorescent images and quantification of the incorporation of EdU in chondrocytes cultured without (Ctrl) and with CD44 inhibition (CD44i) in ‘low’ modification hydrogels at day 7. (scale bar = 100µm, low Ctrl: *n* = 23 ROIs, *N* = 3; low CD44: *n* = 27 ROIs, *N* = 3; high Ctrl: *n* = 23 ROIs, *N* = 3) (c) Representative fluorescent images (dashed line outlines nuclei) and quantification of Sox9 nucleus‐to‐cytoplasm (NC) ratio of chondrocytes cultured without (Ctrl) and with CD44 inhibition (CD44i) in ‘low’ hydrogels at day 7. (scale bar = 10µm, low Ctrl: *n* = 138 cells, *N* = 4; low CD44i: *n* = 110 cells; *N* = 4, high‐Ctrl: *n* = 124 cells; *N* = 4). (d) Representative fluorescent images (dashed lines outline nuclei) and quantification of Sox9 nucleus‐to‐cytoplasm (NC) ratio of chondrocytes pre‐incubated with HA and cultured in ‘low’ and ‘high’ hydrogels at day 7 (scale bar = 10 µm; low HA: n = 124 cells, N = 3; high HA: n = 123 cells, N = 3). Grey boxes indicate the median and interquartile range of the corresponding controls. (e) Representative fluorescent images (dashed lines outline nuclei) and quantification of Sox9 nucleus‐to‐cytoplasm (NC) ratio of chondrocytes cultured in ‘low’ and ‘high’ NorAlg hydrogels at day 7 (scale bar = 10 µm; low NorAlg: n = 74 cells, N = 2; high NorAlg: n = 75 cells, N = 2). N = number of independent experiments. (a–e) *N* = number of independent experiments, ^****^ *p* < 0.0001, ^***^ *p* < 0.001, ^*^ *p* < 0.05, ns: not significant by the Kruskal–Wallis test with Dunn's multiple comparisons test. Box plots = median, interquartile range, and minimum–maximum values.

Given that the degree of hydrogel modification altered cell fate, we next investigated whether differences in cellular recognition of the hydrogel contribute to this response. Hyaluronic acid interacts with the cell surface receptor CD44 [28], which regulates chondrogenic differentiation [29, 30], and whose engagement depends on the degree of HA modification [31, 32]. Thus, to investigate how CD44–hydrogel interactions direct chondrocyte fate, we performed a series of perturbation studies, including treatment with a function‐blocking CD44 antibody (Figure 3a(i)), pre‐incubation with unmodified HA (Figure 3a(ii)), and culture within an alginate hydrogel system that does not rely on CD44‐ligand interactions (Figure 3a(iii)).

First, to inhibit CD44 signaling, chondrocytes were incubated with the function‐blocking CD44 antibody for 1 h before encapsulation and cultured in CD44 antibody containing media throughout the first 3 days of the 7‐day culture period (Figure 3a(i)). Within ‘low’ hydrogels, blocking CD44 signaling induced an almost twofold increase in the number of EdU‐positive cells (49% ± 8%) at day 7, which is similar to the number of EdU‐positive cells in ‘high’ hydrogels without CD44 inhibition (Ctrl, Figure 3b). In addition, blocking CD44 signaling significantly decreased Sox9 nuclear staining, resulting in a reduction of the Sox9 nuclear‐to‐cytoplasmic ratio from 1.79 ± 0.41 (Ctrl) to 1.55 ± 0.29 (CD44i), which is similar to cells in ‘high’ hydrogels without CD44 inhibition (Figure 3c). Interestingly, COL2A1/COL1A1 and ACAN/VCAN ratios were only slightly reduced, with no changes in cell aspect ratios upon CD44 inhibition in ‘low’ hydrogels (Figure S6). Longer CD44 inhibition and culture times may be required to see significant downregulation in chondrogenic gene expression and cell elongation. Note that CD44 inhibition had little influence on EdU incorporation and Sox9 nuclear‐to‐cytoplasmic ratios in ‘high’ hydrogels (Figure S7), indicating that the initial interaction with ‘high’ hydrogels prevents cell proliferation and chondrogenic differentiation. Next, we asked whether pre‐incubating cells with unmodified HA may compensate for differences in HA availability resulting from norbornene modification. Indeed, HA pre‐incubation eliminated the difference in SOX9 nuclear localization between ‘low’ and ‘high’ hydrogels and increased SOX9 nuclear‐to‐cytoplasmic ratio relative to untreated controls (Figure 3d). These findings indicate that the initial interactions of embedded cells with HA regulate downstream differentiation and further support the conclusion that norbornene modification reduces HA availability in both hydrogel formulations. Finally, we isolated the effect of residual free norbornene groups using norbornene‐modified alginate (NorAlg) hydrogels with ‘low’ and ‘high’ degrees of modification. Chondrocytes cultured in ‘low’ and ‘high’ NorAlg hydrogels showed similar SOX9 nuclear‐to‐cytoplasmic ratios (Figure 3e). These findings suggest that residual free norbornene groups alone are insufficient to explain the differences in SOX localization observed between ‘low’ and ‘high’ NorHA hydrogels. Taken together, these findings support a model in which the degree of norbornene modification regulates chondrocyte fate by altering HA availability and CD44 engagement.

### 2.4 Specific nECM Components are Spatially Heterogeneous at the Cell‐Hydrogel Interface

**FIGURE 4:**
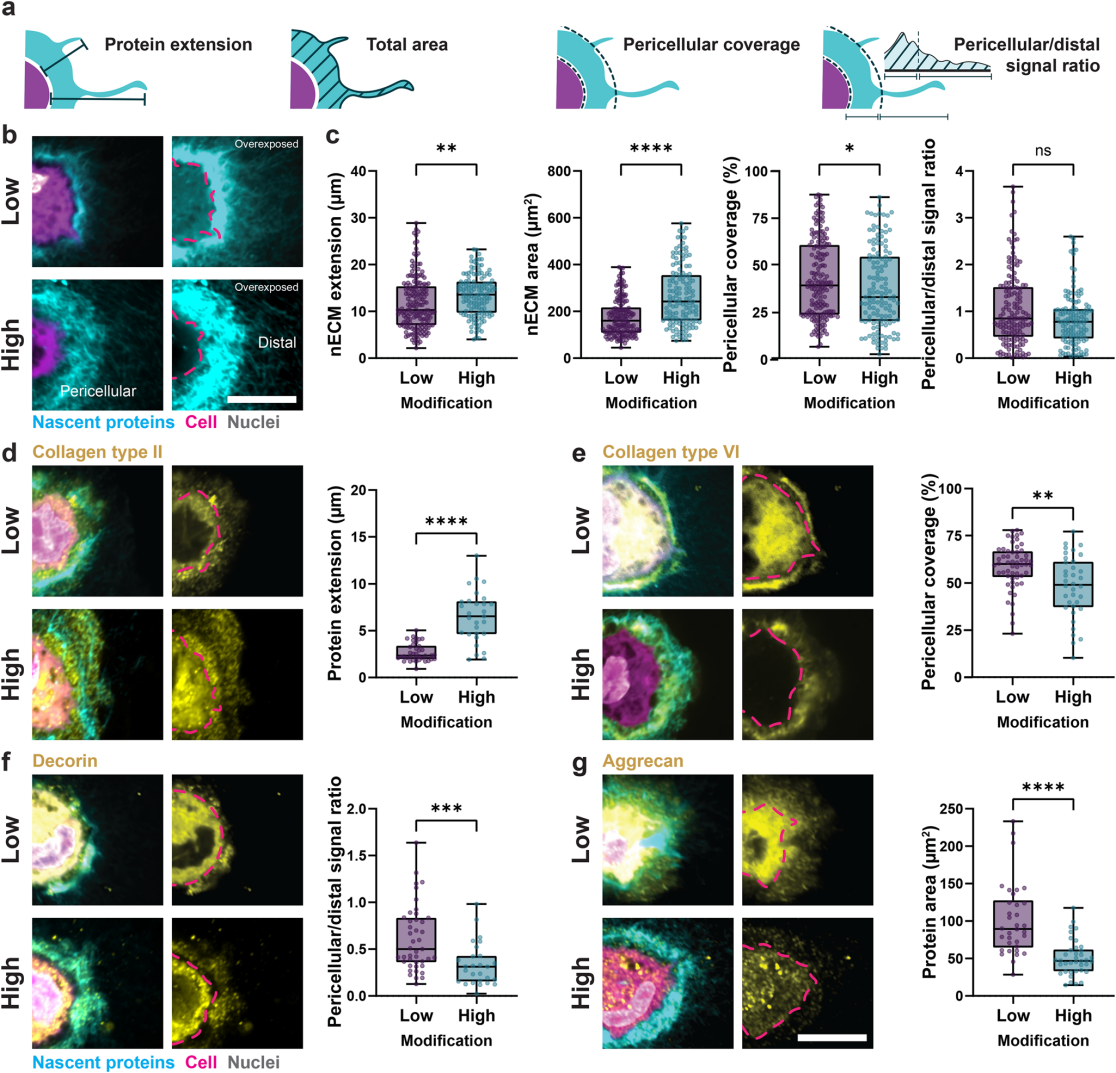
Hydrogel modifications induce differences in spatial organization of nECM and chondrogenic proteins. (a) Schematics illustrating the selected image quantifications to describe the spatial properties of nECM and chondrogenesis‐associated proteins: (i) matrix/protein extension, (ii) matrix/protein area, (iii) pericellular coverage, and (iv) pericellular‐to‐distal integrated signal ratio. (b) Representative fluorescent images of nECM in ‘low’ and ‘high’ modification hydrogels at day 7. Overexposed images are shown to visualize the distal nECM region (scale bar = 10 µm). (c) Quantification of nECM spatial features, including protein extension (low: n = 174 cells; high: n = 133 cells), total area (low: n = 174 cells; high: n = 133 cells), pericellular coverage (low: n = 153 cells; high: n = 133 cells), and the pericellular‐to‐distal integrated signal ratio (low: n = 166 cells; high: n = 133 cells). (d) Representative immunofluorescent images of collagen type II co‐stained for nECM in ‘low’ and ‘high’ modification hydrogels at day 7 and quantification of collagen type II protein extension (low: n = 38 cells; high: n = 29 cells). (e) Representative immunofluorescent images of collagen type VI co‐stained for nECM in ‘low’ and ‘high’ modification hydrogels at day 7 and quantification of collagen type VI pericellular coverage (low: n = 56 cells; high: n = 36 cells). (f) Representative immunofluorescent images of decorin co‐stained for nECM in ‘low’ and ‘high’ modification hydrogels at day 7 and quantification of decorin pericellular‐to‐distal integrated signal ratio (low: n = 43 cells; high: n = 27 cells). (g) Representative immunofluorescent images of aggrecan core protein co‐stained for nECM in ‘low’ and ‘high’ modification hydrogels at day 7 and quantification of aggrecan core protein area (low: n = 35 cells; high: n = 38 cells). Additional image quantifications for each protein are in Figure S9. (a–g) ^****^ *p* < 0.0001, ^***^ *p* < 0.001, ^**^ *p* < 0.01, ^*^ *p* < 0.05, ns: not significant by two‐tailed Mann–Whitney test. Box plots = median, interquartile range, and minimum–maximum values.

Given that initial cell‐hydrogel interactions modulated chondrocyte fate, we next investigated how these interactions affected the spatial organization of the nECM, including proteins associated with chondrogenic matrix formation. To quantify these features, we developed a custom Python‐based image‐analysis pipeline that measured (i) nECM/protein extension from the cell surface, defined as the average distance of the signal into the hydrogel, (ii) total nECM/protein area, (iii) pericellular coverage, defined as the fraction of the pericellular region occupied by protein signal, and (iv) the ratio of pericellular to distal integrated signal (Figure 4a).

Using confocal microscopy, nECM images consistently showed two spatially distinct domains, including a dense pericellular shell and a diffuse, often fibrous distal matrix (Figure 4b). Overexposure of the nECM stain revealed fibrillar ECM that extended much further into the surrounding hydrogel. This is consistent with previous studies in agarose hydrogels [31]. As expected, nECM in ‘high’ hydrogels showed increased nECM area, which was aligned with an increase in nECM extension from the cell surface (Figure 4c). Interestingly, nECM in ‘high’ hydrogels showed a decrease in pericellular coverage with little difference in the pericellular‐to‐distal integrated signal ratio (Figure 4c). This suggests that initial cell‐hydrogel interactions alter the structural organization of the deposited nECM.

Although global nECM labeling showed the overall amount and spatial distribution, it provides limited information about the identity of individual matrix components and their organization within the pericellular and distal extracellular regions. We therefore analyzed key chondrogenic ECM proteins, including collagen type II, collagen type VI, aggrecan, and decorin [33], which showed distinct spatial and structural patterns as supported by UMAP clustering (Figure S8). More specifically, collagen type II showed little fibrillar extension into the hydrogel but maintained increased protein extension from the cell surface in the ‘high’ compared to ‘low’ hydrogels (Figure 4d). Similarly, collagen type VI, a pericellular collagen around chondrocytes [34], was restricted to the immediate pericellular interface in both groups but with a reduction in the pericellular coverage for cells in ‘high’ compared to ‘low’ hydrogels (Figure 4e). These findings indicate that collagens are spatially heterogeneous, and their patterns additionally depend on the initial cell‐hydrogel interactions. Beyond collagens, proteoglycans are also an important component of the chondrogenic ECM. Thus, we next stained for Decorin, an important proteoglycan that regulates collagen fibrillogenesis and matrix micromechanics [35, 36]. Decorin remained largely confined to the pericellular nECM region but with a lower pericellular‐to‐distal signal ratio for cells in ‘high’ hydrogels following nECM trends (Figure 4f). In contrast, staining for the core protein of aggrecan, the major proteoglycan in articular cartilage [33], showed a relatively thick layer within ‘low’ hydrogels but a nearly twofold reduction in protein area within ‘high’ hydrogels (Figure 4g). Additional protein features followed similar trends as observed for nECM stainings (Figure S9). Overall, these observations suggest that both cell‐hydrogel interactions and protein‐hydrogel interactions may regulate the deposition and spatial distribution of nECM components throughout the hydrogel. For example, protein‐specific properties such as molecular size and supramolecular organization may shape their localization within pericellular and extracellular regions. In addition, it is possible that early differences in cell state may alter ECM assembly that influences the retention and spatial organization of newly secreted matrix components. However, further studies are needed to define the relative contributions of molecular‐scale hydrogel properties, ECM assembly, and temporal feedback between cells and their evolving matrix.

### 2.5 Hydrogel Modifications Induce Differential nECM Protein Expression

**FIGURE 5:**
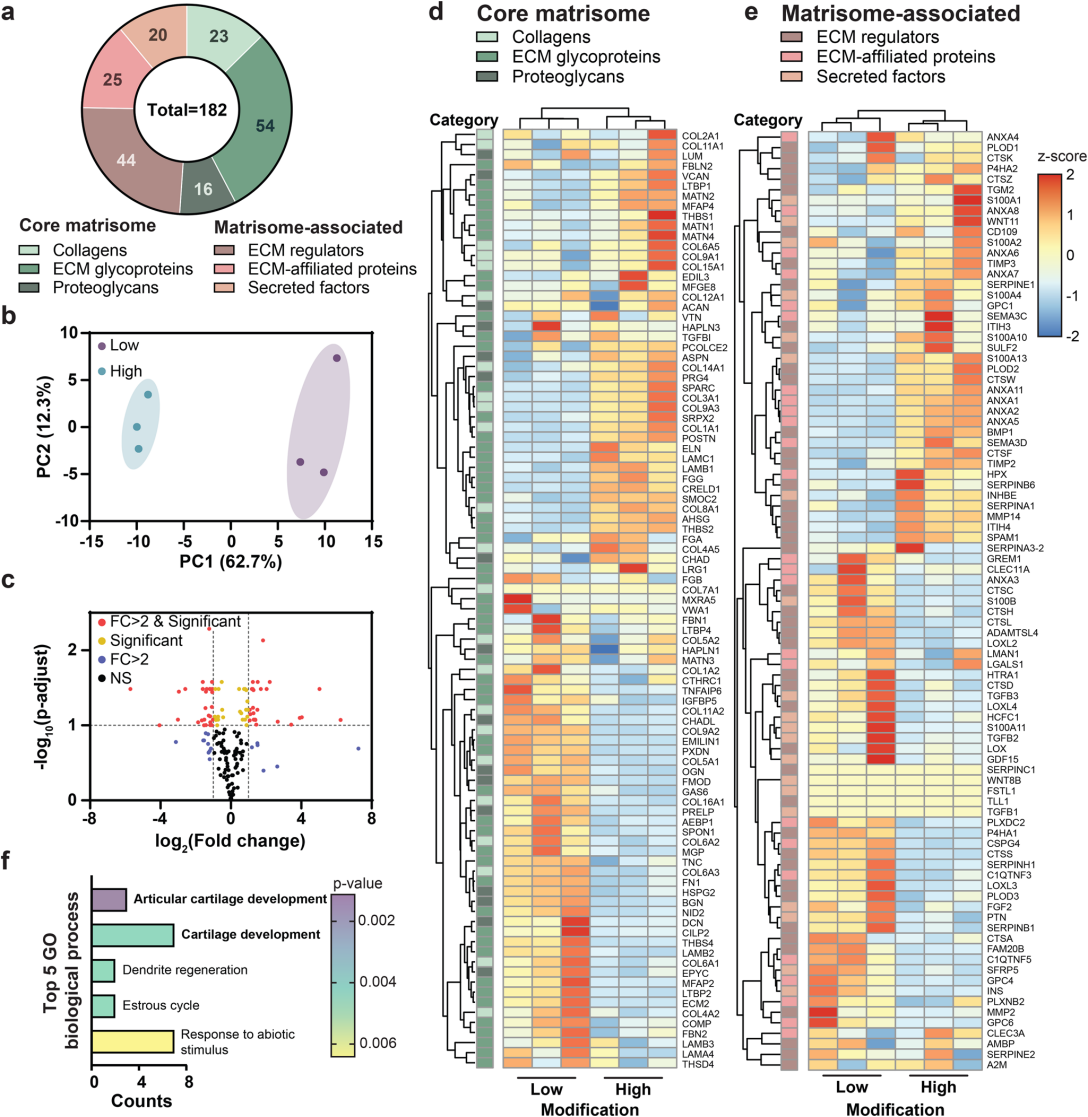
Hydrogel modifications induce differential expression of specific nECM proteins. (a) Representative pie charts showing the distribution of core matrisome proteins (ECM glycoproteins, collagens, and proteoglycans) and matrisome‐associated proteins (ECM regulators, ECM‐affiliated proteins, and secreted factors) deposited by chondrocytes in ‘low’ and ‘high’ hydrogels at day 7. (b) Principal component analysis (PCA) plot of core matrisome and matrisome‐associated proteins deposited by chondrocytes in low‐ and high‐modification hydrogels (*N* = 3 independent experiments). (c) Volcano plot showing differential expression of core matrisome and matrisome‐associated proteins deposited by chondrocytes in high versus low modification hydrogels at day 7. Red dots indicate proteins with differential expression levels and high fold changes (|FC| > 2 and adjusted p < 0.1); yellow dots indicate proteins with only significant by adjusted p (adjusted p < 0.1, |FC| ≤ 2); blue dots indicate high fold change only without significance (|FC| > 2, adjusted p ≥ 0.1); black dots indicate non‐significant proteins changes (|FC| ≤ 2 and adjusted p ≥ 0.1). (*N* = 3 independent experiments). Heatmaps of (d) core matrisome protein expression and (e) matrisome‐associated proteins in low and high modification hydrogels at day 7. Rows show individual proteins grouped by hierarchical clustering analysis: columns show the 3 different samples with expression levels (z‐score by rows) shown as color intensity from negative z‐scores (blue) to positive z‐scores (red/yellow). The left color bar denotes protein categories: core matrisome (ECM glycoproteins, collagens, proteoglycans) and matrisome‐associated proteins (ECM regulators, ECM‐affiliated proteins, and secreted factors). (f) Top 5 significant Gene Ontology (GO) enrichment pathways with p < 0.05 of biological process, including articular cartilage development, cartilage development, dendrite regeneration, estrous cycle, and response to abiotic stimulus. Counts indicate the number of proteins significantly enriched in each pathway. *N* = number of independent experiments.

Next, we asked whether these distinct spatial features of chondrogenic ECM proteins are reflected in difference of the overall composition of the nECM. To address this, we performed unbiased bulk proteomic analysis to identify and quantify expression of nECM proteins in ‘low’ and ‘high’ hydrogel. Given the low abundance of nECM proteins within all proteins, we enriched the samples for ECM proteins prior to mass‐spectrometry by decellularizing the hydrogels followed by collecting the ECM proteins in high‐concentration urea (8M) [6, 9]. Next, we used the Matrisome Analyzer [37] and classified 182 proteins as matrisome components, including the core matrisome (93 total: collagens (23), glycoproteins (54), and proteoglycans (16)) and matrisome‐associated proteins (89 total: ECM regulators (44), ECM‐affiliated proteins (22), and secreted factors (22) (Figure 5a). Principal component analysis (PCA) for the nECM component abundances revealed clear separation of the proteins within ‘low’ and ‘high’ hydrogels along PC1 (62.7% of the total variance) and PC2 (12.3% of the variance, Figure 5b). This tight clustering within each group indicates low technical variability and reproducible measurements across independent experiments. Differential expression was determined using an adjusted *p*‐value (*p adj*) threshold of 0.1 (FDR (False Discovery Rate), Benjamini–Hochberg) to maintain sensitivity while controlling for multiple testing in exploratory proteomic datasets. Among the 182 identified matrisome proteins, 55 were significantly different (|fold change| > 2 and *p adj* < 0.1), while 17 showed > twofold change only and 26 showed *p adj* < 0.1 only (Figure 5c). Thus, 30% of the identified matrisome proteins showed a significant and more than twofold differential expression between ‘low’ and ‘high’ groups. Building upon these results, we next compared specific protein expressions between ‘low’ and ‘high’ groups. Within the core matrisome, several core proteins, including collagen type VI (COL6A3), biglycan (BGN), osteoglycin (OGN), and decorin (DCN, non‐significant), were upregulated in ‘low’ compared to ‘high’, suggesting that the nECM is chondrogenic. In contrast, collagen type I (COL1A1), versican (VCAN), and collagen type III (COL3A1) were upregulated in ‘high’ hydrogels, suggesting upregulation of genes linked to fibrotic ECM remodeling (Figure 5d and Table S1). Within the matrisome‐associated proteins, the differential expression of lysyl oxidases (LOXL2, LOXL3 and LOXL4), matrix metalloproteinases (MMP14), and tissue inhibitor of metalloproteinases (TIMP2 and TIMP3) indicates differences in the regulation of nECM crosslinking, degradation and remodeling between ‘low’ and ‘high’ hydrogels (Figure 5e and Table S1). Gene Ontology (GO) analysis performed on the 55 differentially expressed matrisome proteins with the full matrisome (∽1,000 genes) [38] as the reference background revealed enrichment in articular cartilage development (GO:0061975) and cartilage development (GO:0051216). Enriched protein includes biglycan (BGN), epiphycan (EPYC), osteoglycin (OGN), LOXL2, Matrix Gla Protein (MGP), COL1A1, and COL3A1 (Figure 5f). These enrichments align with gene expression data (Figure 2i) and indicate that the nECM compositional differences in ‘low’ hydrogels are associated with cartilage developmental processes. It further aligns with previous studies showing that low‐modification NorHA hydrogels increase cartilage tissue formation in long‐term in vitro culture [20].

Taken together, these findings connect changes in nECM deposition and chondrogenic cell fate with distinct changes in the nECM matrisome, suggesting a direct relationship between hydrogel modifications, nECM composition, and cell fate.

### 2.6 Cell‐nECMs Interactions Determine Cell Fate

**FIGURE 6:**
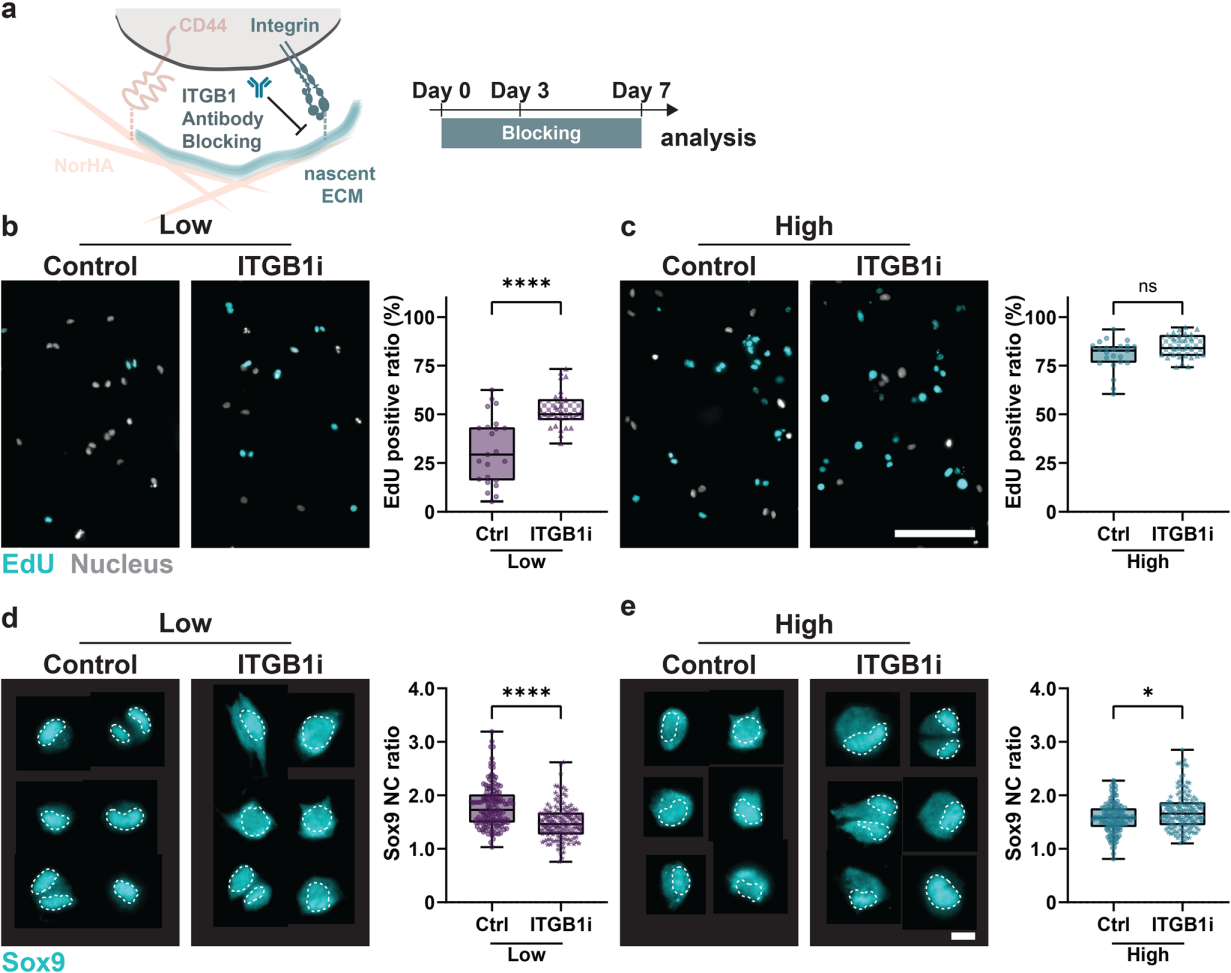
Cell–nECM interaction via integrin β1 regulates chondrogenic differentiation. (a) Schematic and experimental timeline illustrating integrin β1 (ITGB1) function‐blocking antibody treatment throughout the 7‐day culture period. (b) Representative fluorescence images and quantification of EdU incorporation in chondrocytes cultured in low hydrogels without (Ctrl) or with integrin β1 inhibition (ITGB1i) at day 7 (low‐Ctrl: n = 23 ROIs, N = 3; low‐ITGB1i: n = 30 ROIs, N = 3; scale bar = 100 µm). (c) Representative fluorescence images and quantification of EdU incorporation in chondrocytes cultured in high hydrogels without (Ctrl) or with integrin β1 inhibition (ITGB1i) at day 7 (high‐Ctrl: n = 23 ROIs, N = 3; high‐ITGB1i: n = 27 ROIs, N = 3; scale bar = 100 µm). (d) Representative Sox9 immunofluorescence images (dashed lines outline nuclei) and quantification of the Sox9 nuclear‐to‐cytoplasmic (N/C) ratio in chondrocytes cultured in low hydrogels without (Ctrl) or with integrin β1 inhibition (ITGB1i) at day 7 (low‐Ctrl: n = 138 cells, N = 4; low‐ITGB1i: n = 133 cells, N = 4; scale bar = 10 µm). (e) Representative Sox9 immunofluorescence images (dashed lines outline nuclei) and quantification of the Sox9 nuclear‐to‐cytoplasmic (N/C) ratio in chondrocytes cultured in high hydrogels without (Ctrl) or with integrin β1 inhibition (ITGB1i) at day 7 (high‐Ctrl: n = 124 cells, N = 4; high‐ITGB1i: n = 138 cells, N = 4; scale bar = 10 µm). N indicates the number of independent experiments. (b–e) ^****^ *p* < 0.0001, ^*^ *p* < 0.05, ns: not significant by two‐tailed Mann–Whitney test. Box plots = median, interquartile range, and minimum–maximum values.

Given that cell‐hydrogel interactions altered both the composition and spatial organization of the nascent matrisome, we next asked whether this deposited nECM itself contributes to the maintenance of chondrogenic phenotypes. Because cells interact with nECM via integrins, we treated cells with a function‐perturbing antibody against integrin β1 (ITGB1i), a major integrin subunit involved in cell adhesion to diverse ECM proteins [39]. Our previous studies showed that cells deposit nECM within a few hours [4]. We therefore blocked cell‐nECM interactions immediately after embedding and for the entire cell culture period (Figure 6a). The treatment with ITGB1i for 7 days had minimal effect on nECM deposition, and IgG isotype controls showed similar differentiation potential to untreated cells (Figure S10).

Interestingly, inhibition of ITGB1 in ‘low’ hydrogels resulted in an almost twofold increase in EdU incorporation with little changes between Ctrl (Figure 6b). In contrast, no differences were observed for ITGB1i‐treated cells in ‘high’ hydrogels (Figure 6c). This suggests that blocking interactions between the cell and chondrogenic nECM in ‘low’ hydrogels leads to similar high cell proliferation in ‘high’ hydrogels. Notably, blocking ITGB1 of cells in ‘low’ hydrogels induced a significant decrease in Sox9 nuclear staining and nuclear‐to‐cytoplasmic ratio to the same levels as observed in ‘high’ hydrogels (Figure 6d). This shows that cellular interaction with the nECM in ‘low’ hydrogels provide the pro‐chondrogenic signals that are required to maintain cell fate and that this goes beyond the initial hydrogel modifications. In contrast, blocking ITGB1 in ‘high’ hydrogels increased Sox9 nuclear staining and nuclear‐to‐cytoplasmic translocation (Figure 6e), indicating a rescue of cell differentiation. The ability to restore Sox9 cytoplasmic‐to‐nuclear transition by disrupting cell‐nECM interactions suggests that nECM in ‘high’ hydrogels promotes de‐differentiation, highlighting the instructive role of nECM. Notably, the ITGB1 inhibitor used here targets the β1 subunit and therefore may disrupt multiple β1−containing heterodimers expressed by chondrocytes [39]. Because β1‐containing integrins and CD44 both mediate interactions with the developing pericellular matrix, we also tested their combined contribution to Sox9 regulation. In ‘high’ hydrogels, simultaneous inhibition of CD44 and ITGB1 produced no additional increase in nuclear Sox9 relative to ITGB1 inhibition alone (Figure S11a), consistent with a shared or saturable response. In contrast, in ‘low’ hydrogels, simultaneous inhibition restored nuclear Sox9 expression despite the reduction caused by inhibiting either receptor individually (Figure S11b), indicating an antagonistic or compensatory interaction. These findings demonstrate some crosstalk between CD44 and β1‐containing integrins. However, additional studies, such as inhibition of β1‐containing integrin heterodimers, measurement of integrin activation, or selective siRNA‐mediated knockdown will be required to establish whether CD44 regulates β1‐integrin activation or whether the receptors act through parallel pathways.

## 3 Outlook

**FIGURE 7:**
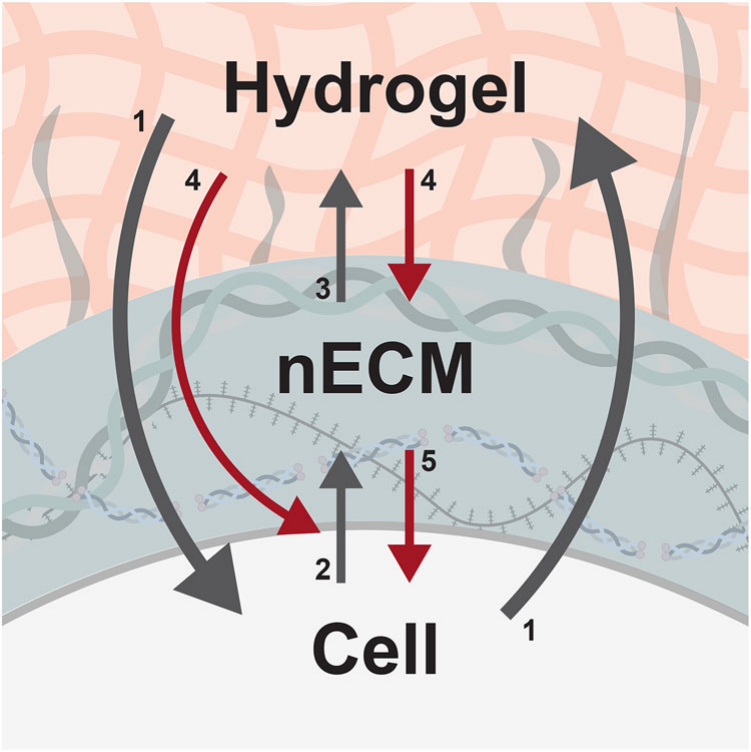
Tri‐directional interplay among cells, hydrogels and their nECM. Schematic illustrating previous findings (grey arrows), including the traditional bi‐directional interplay between cells and the ECM or engineered hydrogels (**1**, Bissel 1982^2^), and recent work that showed the rapid deposition of nECM after embedding cells into engineered hydrogels (**2**, McLeod 2016; Loebel 2019; Cha 2024 [4, 6, 31]) that interpenetrates into the existing hydrogel (**3**, Loebel 2020^8^). This work (red arrows) demonstrates that hydrogel modifications direct nECM deposition and accumulation (**4**) and induce cell fate decisions (**5**).

Previous studies have focused on engineering hydrogels with tunable mechanical and biochemical properties to mimic cell‐ECM interactions and instruct cell behavior [40]. More recent work has revealed that cells rapidly deposit nECM upon embedding, creating a dynamic interface between cells and the engineered hydrogels [4, 6, 31]. Yet, the complex interactions between cells, hydrogels, and the nECM have not been well studied. Using hydrogels with variable chemical modifications and embedded chondrocytes, we establish a new framework describing a tri‐directional interplay among cells, hydrogels, and the deposited nECM (Figure 7). Specifically, we found that low hydrogel norbornene modifications led to reduced deposition and accumulation of nECM. Blocking cellular interactions via integrin β1 inhibition reduced their chondrogenic cell fate. In contrast, high norbornene modifications increased nECM accumulation but with less‐chondrogenic compositional signatures that promoted proliferation over differentiation. Notably, blocking cellular interactions with ‘high’ nECM rescued the chondrogenic cell fate. This finding highlights that the observed phenotypic changes are not governed solely by the hydrogel itself; rather, the deposited nECM may act as a cell‐instructive microenvironment that subsequently directs cell fate. Thus, our study extends Mina Bissell's pioneering concept of bidirectional communication between cells and their ECM or engineered hydrogel [2, 3] by revealing the nECM as a third regulatory player that actively governs cell fate decisions in 3D hydrogel systems. The current study does not establish the mechanisms by which norbornene modifications regulate nECM deposition or determine whether the response is due to material properties that are not captured by conventional characterization methods. Further work is needed to identify the underlying mechanisms. An additional limitation is the focus on norbornene modifications, although these are widely used in the field [41, 42, 43]. Whether other chemical modifications similarly influence cell function remains unknown. To establish the broader applicability of these findings, systematic studies of hydrogel backbone chemistry and the type and degree of modifications are required. In addition, appropriate controls that disentangle these effects from changes in other material properties are needed.

In this work, we selected chondrocytes as a well‐characterized cell source that responds to hyaluronic acid backbone signaling and is a robust ECM producer [44]. Our previous studies showed that hydrogel properties regulate nECM thickness of bovine mesenchymal stromal cells [8], implying that this framework may be generalizable across cell types. We and others have also demonstrated nascent ECM deposition by adipose‐derived stem cells [45], muscle stem cells [7], lung epithelial cell progenitors [11], tendon fibroblasts [46, 47], and glioblastoma cells [6]. However, additional studies are required to further support these claims across different cell types and hydrogel platforms. Furthermore, several studies have incorporated cell‐adhesive moieties and degradable hydrogel crosslinkers, which may additionally regulate nECM deposition and interpenetration with the hydrogel [13, 48]. Future work incorporating peptides like RGD or collagen‐mimetic binding peptides and the use of enzymatically or hydrolytically‐degradable peptide crosslinkers will be critical to further expand and validate this tri‐directional framework across diverse biomaterial systems.

Taken together, our work demonstrates that hydrogel chemical modifications guide cell fate through the deposition and accumulation of nECM and subsequent cell‐nECM interactions. This tri‐directional framework provides a new lens through which to understand cell‐biomaterial interactions. It further offers design principles for engineering biomaterials that harness nECM as an instructive intermediary to direct desired cellular outcomes.

## 4 Experimental

### 4.1 Hydrogel synthesis

NorHA was synthesized via a 4‐(4,6‐dimethoxy‐1,3,5‐triazin‐2‐yl)‐4‐methylmorpholinium chloride (DMTMM)‐mediated aqueous coupling reaction adapted from previously reported methods [21]. Sodium hyaluronate (Lifecore Biomedical, MW ∽68.1 kDa) was dissolved at 1% (w/v) in 0.1 M MES buffer (pH 5.5). DMTMM (TCI, >98%) and 5‐norbornene‐2‐methylamine (Nor, TCI, >98%) were added at defined molar ratios to tune the degree of modification. For low modification, a 1:1:3 HA:DMTMM:Nor ratio was reacted for 24 h. For mid, 1:3:2 was used for 24 h. For high, the same 1:3:2 ratio was used with a second addition of DMTMM and Nor after 24 h, continuing for 48 h total. Following the reaction, NorHA was precipitated with saturated NaCl and ethanol, then resuspended in Milli‐Q water and dialyzed for three days using 6–8 kDa tubing against 0.25 g/L DPBS (Gibco). The final product was frozen, lyophilized, and stored at −20 °C. To determine the degree of norbornene modification, we performed proton nuclear magnetic resonance (^1^H NMR) spectroscopy. Modified hyaluronic acid products were dissolved in deuterium oxide at 7 mg/mL, and spectra were acquired using a 600 MHz NEO400 spectrometer (Bruker). The modification degree was calculated from the ratio of integrated signal intensities corresponding to backbone protons of hyaluronic acid and vinyl protons of the norbornene groups. (Figure S12). Baseline correction and spectral analysis were conducted in MestReNova (v15.1.0, Mestrelab Research).

### 4.2 Hydrogel Formation

Lyophilized NorHA was sterilized using a UV oven (Tool Klean) for 30 min. Hydrogels were prepared at 2 wt.% by dissolving the sterilized NorHA in buffer (pH 7.5) containing 50 mM HEPES (Gibco) and 1 mg/mL phenol red (Remel). The photoinitiator lithium phenyl‐2,4,6‐trimethylbenzoylphosphinate (LAP, Arkema Sartomer) was added at a final concentration of 1.70 mM, and dithiothreitol (DTT, Sigma–Aldrich) was included as the crosslinker at designated concentrations ranging from 0.13 to 13 mM. The precursor solution was then exposed to UV light (OmniCure, 5 mW/cm^2^) for 3 min to initiate gelation. For swelling measurements, hydrogels were incubated in PBS at 37°C, and wet weights were recorded at 0, 3, 6, 12, 24, 48, and 72 h. Swelling ratios were calculated by normalizing the wet weight at each time point to the initial wet weight immediately after gelation (0 h).

### 4.3 NorAlg Synthesis and Hydrogel Formation

Norbornene‐modified alginate (NorAlg) was synthesized using the same DMTMM‐mediated coupling chemistry described for NorHA. Briefly, sodium alginate (Sigma–Aldrich) was reacted with DMTMM and 5‐norbornene‐2‐methylamine in 0.1 M MES buffer (pH 5.5) at room temperature for 24 h. Low and high modification conditions were generated using Alg:DMTMM:Nor molar ratios of 2:1:3 and 1:1:3. Following purification using dialysis against deionized water for 7 days, polymers were frozen, lyophilized, and stored at −20°C. The degree of modification was determined by ^1^H NMR spectroscopy using 2 mM Dimethylformamide (DMF, Sigma–Aldrich) as an internal standard to account for the broad alginate backbone signal. The amount of norbornene was quantified from the integrated vinyl proton peaks relative to the formyl proton peak of DMF, and the degree of modification was calculated based on polymer mass (Figure S13a). NorAlg hydrogels were prepared at 1.2 wt.% with 1.70 mM LAP and 1 mM DTT and photo‐crosslinked under UV light (5 mW/cm^2^, 3 min) to obtain ∽ 5 kPa hydrogels (Figure S13b)

### 4.4 Mechanical Characterization

For axial compression testing, NorHA hydrogels were cast in cylindrical molds (5 mm diameter) and subjected to unconfined compression using a Discovery HR‐30 Hybrid Rheometer (TA Instruments) at room temperature with a constant compression rate of 20 µm/s. Young's modulus was calculated from the linear region of the stress–strain curve between 10% and 20% strain. For stress‐relaxation testing, NorHA hydrogels were cast in 24‐well plates, compressed at 20 µm/s to 15% strain, and held at constant deformation while stress relaxation was recorded for 1,000 s. The storage modulus of NorAlg hydrogels was measured with a 20‐mm cone‐and‐plate geometry. Hydrogel precursors were photocrosslinked in situ during an 1‐Hz oscillatory time sweep, and the plateau storage modulus (G′) was used for analysis.

### 4.5 Cell culture, Encapsulation and Antibody Blocking

Primary chondrocytes were isolated from juvenile bovine articular cartilage as previously described. Briefly, femoral condyles from juvenile bovine knees (6 months old, purchased from Research 87) were dissected to collect articular cartilage. The tissue was digested in 1 mg/mL type II collagenase (Worthington Biochemical) in DPBS for 20 h at 37 °C in a humidified incubator with 5% CO_2_. The resulting cell suspension was filtered through a 70 µm cell strainer to remove debris. Isolated chondrocytes were expanded for one passage on tissue culture‐treated dishes at a seeding density of 10,000 cells/cm^2^ in high‐glucose DMEM (Gibco) supplemented with 10% fetal bovine serum (FBS, Corning), 1% penicillin–streptomycin (Gibco), and 1% sodium pyruvate (Gibco). Expanded cells were encapsulated in NorHA hydrogels at a density of 5 million cells/mL. Cell‐laden hydrogels were cultured for 7 days in chondrogenic‐azidohomoalanine (AHA) medium composed of glutamine, L‐methionine, and L‐cystine‐free high‐glucose DMEM (Gibco), supplemented with 0.1 µM dexamethasone (Sigma–Aldrich), 4 mM GlutaMAX (Gibco), 0.201 mM L‐cystine (Sigma–Aldrich), 50 µM L‐methionine(Sigma–Aldrich), 100 µg/mL sodium pyruvate, 1.25 mg/mL bovine serum albumin (BSA, Sigma–Aldrich), 0.1% ITS+ premix (Gibco), 50 µg/mL ascorbate‐2‐phosphate(Sigma–Aldrich), 40 µg/mL L‐proline (Sigma–Aldrich), and 1% penicillin–streptomycin–amphotericin. Media were further supplemented with 10 ng/mL Transforming Growth Factor Beta (TGFβ)‐3 (R&D system) and 50 µM L‐AHA (Vector Lab).

For perturbation studies, media were supplemented with either an anti‐CD44 antibody (DSHB, H4C4, 2.5 µg/mL, day 0–3) or anti‐integrin β1 antibody (anti‐ITGB1, DSHB, AIIB2, 2.5 µg/mL, day 0–7). For CD44 blocking studies, cells were additionally incubated in DPBS with anti‐CD44 for 1 h prior to encapsulation [49]. Mouse IgG (R&D Systems, MAB002, 2.5 µg/mL) and rat IgG (R&D Systems, 6‐001‐F, 2.5 µg/mL) were used as isotype controls for the anti‐CD44 and anti‐integrin β1 treatments (Figure S10). For unmodified hyaluronic acid (HA) treatment, cells were preincubated in 2 wt.% HA in PBS for 30 min, pelleted by centrifugation, and immediately re‐encapsulated in hydrogels.

### 4.6 Cell Proliferation Assays

For divided cell quantification, cells were stained with Hoechst 33342 (Invitrogen) and CellMask Deep Red (Invitrogen) and fixated with 4% paraformaldehyde. Cells were classified as “divided” when either multiple nuclei were observed within a single continuous membrane boundary, or two closely apposed cells remained physically connected, consistent with recently divided daughter cells. For EdU incorporation assays, 5 µM EdU was added to chondrogenic AHA media, and incorporated EdU was labeled after fixation using the Click‐&‐Go Cell Reaction Buffer Kit (Vector Labs, CCT‐1263) and AZDye 647 Azide (Click Chemistry Tools, 1482‐1) via copper‐catalyzed click chemistry. Imaging was performed blindly by selecting random regions using a Leica THUNDER microscope (40× objective), acquiring Z‐stacks spanning 100 µm starting from 50 µm below the hydrogel surface. The divided cell ratio was calculated as the number of divided cells divided by the total number of cells, while EdU incorporation was determined by the ratio of EdU‐positive nuclei to total nuclei.

### 4.7 Gene Expression

Total mRNA was isolated from encapsulated cells. Briefly, hydrogels were degraded using 2 mg/mL hyaluronidase (Sigma–Aldrich) by 30 min incubation at 37°C, followed by RNA extraction using the TRIzol‐chloroform method. RNA quality was confirmed by NanoDrop One spectrophotometer (Thermo Fisher Scientific), and RNA concentration was measured using the Qubit RNA High Sensitivity (HS) Assay Kit (Invitrogen, Q32852). cDNA synthesis was performed using the High‐Capacity cDNA Reverse Transcription Kit (Applied Biosystems, Invitrogen, 4368814) according to the manufacturer's protocol. Quantitative PCR (qPCR) was conducted using PowerUp SYBR Green Master Mix (Invitrogen, A25742) on an Applied Biosystems QuantStudio 3 Real‐Time PCR System, with three technical replicates per sample.

Gene expression levels were calculated using the ΔΔCt method, with Ribosomal Protein S18 (RPS18) as the reference gene. Expression levels were normalized to the ‘low’ group. In addition to individual gene expression analysis, COL2A1/COL1A1 and ACAN/VCAN expression ratios were also calculated to assess chondrogenic differentiation and matrix composition [50, 51, 52, 53].

**Table:** 

**Gene**	**Direction**	**Sequence**
ACAN	Forward	5' CAC TGT TAC CGC CAC TTC CC
	Reverse	5' GAC ATC GTT CCA CTC GCC CT
COL1A1	Forward	5' CCAATGGCGCTCCTGGTATT
	Reverse	5' ACCAGGTTCACCGCTGTTAC
COL2A1	Forward	5' CAGGACGGGCAGAGGTATAATG
	Reverse	5' CAGAGGACAGTCCCAGTGTCA
RPS18	Forward	5' GTGGTGTTGAGGAAAGCAGACA
	Reverse	5' TGATCACACGTTCCACCTCATC
SOX9	Forward	5' GCGGAGATTGAAACTGACCT
	Reverse	5' CTCTCCTCCCTCCTGCAAAGA
VCAN	Forward	5' TGCCTACTGCTTTAAACCTAAAC
	Reverse	5' TGGGTGAGACAGTTTCTGCAA

### 4.8 Fluorescence Recovery After Photobleaching (FRAP)

FRAP experiments were performed using a Nikon Eclipse Ti2‐E microscope equipped with a 60× objective. Hydrogel (∽660 µm thick) containing 100 µM fluorescein isothiocyanate (FITC)‐dextran (150 kDa) was incubated in PBS supplemented with 100 µM FITC‐dextran for 24 h to reach equilibrium swelling prior to imaging. A circular region (40 µm in diameter) was photobleached using a 488 nm laser for 5 s, and fluorescence recovery was recorded every 3 s. Recovery curves were analyzed using a custom MATLAB script by fitting an exponential model, (I(t) = A(1‐exp(‐t/τ)), where (I) is the normalized fluorescence intensity, (t) is time, (τ) is the characteristic recovery time, and (A) is the mobile fraction. The effective diffusivity (D_eff_) was calculated as (D_eff_ = ω^2^/4τ), where (ω) is the bleach spot radius [8, 54].

### 4.9 Fibronectin Adsorption

Acellular hydrogels were incubated in serum‐free DMEM containing 30 µg/mL HiLyte 488‐labeled fibronectin (Cytoskeleton Inc.) for 48 h. Hydrogels were imaged using a Leica DMi8 THUNDER widefield microscope equipped with a 25× water‐immersion objective. Mean fluorescence intensity was quantified for each field of view and normalized to hydrogels incubated in serum‐free DMEM without fluorescent fibronectin.

### 4.10 Immunofluorescence Staining, Imaging and Analyses

For nECM labeling, samples were incubated with 30 µM AZDye 488 DBCO (Vector Laboratories) to conjugate the incorporated AHA‐labeled proteins, followed by washing in PBS. For 3D nECM thickness analysis, samples were additionally stained with CellMask Deep Red (Invitrogen, 1:1000) and Hoechst 33342 to label cell boundaries and nuclei, respectively, prior to fixation Samples were fixed with 4% paraformaldehyde. For experiments requiring cryosections, samples were cryoprotected by sequential incubation in 30% sucrose overnight and cryoprotected by sequential incubation in 30% sucrose overnight, followed by 2 h in a 1:1 mixture of sucrose and OCT compound, and 1 h in 100% OCT. Samples were embedded in OCT and placed into a room‐temperature 2‐methylbutane bath (Thermo Fisher Scientific), which was then snap‐frozen by immersion in liquid nitrogen to minimize ice crystal formation. Cryosectioning was performed at −23 °C using a Leica CM3050S cryostat to obtain 10 µm thick slices. Slides were baked at 37 °C for 30 min before storage at −20 °C. Cryosections were blocked in 5% (w/v) BSA for 30 min, incubated with primary antibodies diluted in 2% BSA overnight at 4°C, and then incubated with secondary antibodies in 2% BSA for 1 h at room temperature. DAPI (Invitrogen, 62248, 1:1000) and HCS cytoplasm CellMask Deep Red (Invitrogen, H32721, 1:5000) were included during secondary antibody incubation. Slides were mounted using SlowFade Diamond antifade mountant with DAPI (Invitrogen, S36968). Primary antibodies included SOX9 (NovusBio, NBP2‐24659, 1:100), YAP (Santa Cruz Biotech, sc‐101199, 1:200), Collagen II (DSHB, II‐II6B3, 1:100), Collagen VI (Biosynth, 70R‐CR009X, 1:100), Decorin (Kerafast, ENH077‐FP, 1:100), and Aggrecan (Abcam, ab3778, 1:50). Secondary antibodies included goat anti‐mouse IgG Alexa Fluor 568 (Invitrogen, A11031, 1:200) and goat anti‐rabbit IgG Alexa Fluor 568 (Invitrogen, A11011, 1:200).

Imaging was performed using either a Nikon Eclipse Ti2‐E microscope (60× objective) or a Leica THUNDER microscope (40× objective). The regions of interest were chosen blindly, and mages acquired at the midplane of the cell body.

Calculation of SOX9 and YAP nucleus‐to‐cytoplasm ratios wer calculated for each individual cell. The DAPI channel was used to generate a nuclear mask, while the cytoplasmic region was defined by subtracting the nuclear mask from the CellMask channel. The mean fluorescence intensity of SOX9 or YAP within each region was measured, and the nuclear‐to‐cytoplasmic ratio was calculated by dividing the nuclear intensity by the cytoplasmic intensity.

### 4.11 ECM Imaging and Spatial Analysis

3D nascent matrix thickness was performed following a previously established protocol [9]. Imaging was performed blindly by selecting random regions using a Leica THUNDER microscope (40× objective), acquiring Z‐stacks spanning 100 µm starting from 50 µm below the hydrogel surface. Individual cells were cropped manually, and unbiased image analysis was conducted using an ImageJ macro. Otsu auto‐thresholding was applied to both the nECM and CellMask channels. To isolate the extracellular region of nECM, the CellMask signal was subtracted from the nECM channel. The average thickness of nECM of individual cells was calculated using the BoneJ plugin, while volumes of the cell and nECM were quantified using the 3D Viewer plugin.

Multi‐channel confocal images of cryosectioned samples were analyzed using a custom Python pipeline developed in Visual Studio Code (see https://github.com/loebellab/nascent‐ecm‐cell‐fate.git). ECM and protein images were normalized, Wiener filtered, thresholded, and size filtered. Cell body masks were subtracted to exclude intracellular signal. These masks were used to quantify signal area, extension from cell boundary, pericellular coverage, and the pericellular‐to‐distal signal ratio using physical units (µm or µm^2^). Pericellular and distal regions were defined from radial ECM fluorescence profiles using a 65% threshold relative to the pericellular peak and distal background. Multivariate analysis was performed using 47 quantified spatial features (Table S2). After excluding unsuitable features, skewed features were transformed using the Yeo‐Johnson transformation. Principal Component Analysis (PCA) explaining at least 90% of the variance was retained and used for Uniform Manifold Approximation and Projection (UMAP) visualization of protein spatial organization across ‘low’ and ‘high’ hydrogel groups.

### 4.12 Proteomics and Matrisome Analysis

Protein isolation and nECM enrichment were performed based on a previously described protocol. Briefly, hydrogels were snap‐frozen in liquid nitrogen and subsequently decellularized using 1.5 M potassium chloride (KCl, Sigma–Aldrich) with 0.1% Triton X‐100 (Sigma–Aldrich) in 50 mM Tris‐HCl (Sigma–Aldrich) buffer at pH 8.0 on ice for 6 h in the presence of a protease inhibitor cocktail (cOmplete, Roche). Residual hydrogel and DNA were enzymatically digested overnight at 37 °C using 0.5mg/mL hyaluronidase and DNase (GLPBIO), with continued protease inhibition.

After discarding the supernatant, the pelleted protein was solubilized in 8 M urea (Thermo Fisher Scientific) prepared in 50 mM ammonium bicarbonate buffer (Sigma–Aldrich), followed by acetone precipitation at a 1:4 ratio and overnight incubation at 4 °C. Protein concentration was determined using the BCA assay (Thermo Fisher Scientific, A55864), and 40 µg of protein from each sample was used for downstream analysis.

Samples were labeled with the TMTsixplex Isobaric Label Reagent Set (Thermo Fisher Scientific) and submitted to the University of Michigan Proteomics Core for tandem mass tag (TMT)‐based mass spectrometry on a fee‐for‐service basis. Protein identification and quantification were performed using Proteome Discoverer 3.0. Data were searched against the Bos taurus UniProt database (sp_tr_canonical, TaxID 9913, v2023‐06‐28), allowing dynamic modifications including methionine oxidation (+15.995 Da), deamidation (+0.984 Da, N/Q), and methionine‐to‐azidohomoalanine substitution (−4.986 Da, M). Trypsin was used as the digestion enzyme, and results were filtered at 1% false discovery rate (FDR) for high‐confidence peptide and protein identification.

Annotation of matrisome proteins, including collagens, glycoproteins, proteoglycans, ECM‐affiliated proteins, ECM regulators, and secreted factors, was performed using Matrisome AnalyzeR, based on the bovine matrisome classification and the Matrisome Project database [37, 38, 55]. Within each sample, matrisome protein abundances were normalized by dividing by the mean abundance of all detected matrisome proteins. Differential expressions were assessed by calculating Z‐scores of each protein across samples. Hierarchical clustering was performed on the normalized matrisome subset to generate heatmaps, while principal component analysis (PCA) and volcano plots were constructed based on differential expression data. Gene Ontology (GO) over‐representation analysis was conducted using the WEB‐based GEne SeT AnaLysis Toolkit (WebGestalt, https://www.webgestalt.org/). Differentially expressed matrisome proteins (fold change > 2 and *p‐adjust* < 0.1) were used as the input list, with the full matrisome database serving as the reference background. The analysis was performed using the *Bos taurus* genome as the organism of interest and “Biological Process” as the functional category. Statistical significance was determined by Fisher's exact test, and multiple testing correction was applied using the Benjamini‐Hochberg (BH) method.

### 4.13 Statistical Analysis

Statistical analyses were performed using GraphPad Prism (v10.6) and RStudio (v2025.05.1). Unpaired two‐tailed Student's *t*‐tests with Welch's correction were applied for comparisons between two groups, and one‐way or two‐way ANOVA was used for comparisons among multiple groups. Data visualization was performed using GraphPad Prism. In all figures, *n* represents individual cells or regions of interest (ROIs), and *N* represents independent experiments. Statistical analyses were performed using *n* as the unit of analysis. Experiments were conducted using primary cells from three independent donors, with independent experiments performed using cells derived from these donors across different experimental conditions. Outlier detection was performed using the ROUT method (Q = 1%) implemented in GraphPad Prism. Outliers were removed only for the SOX9 nuclear localization dataset.

## Author Contributions

**J.Y. Liu** conceived and designed the study, performed all experiments, analyzed the data, prepared the figures, and drafted the manuscript. E.M. Plaster, **M. Fan**, D. Ahmed, **A. Roy**, **P. Duran**, **Z. Yang**, **A.E. Velieva** and **P. Panovich** contributed to experimental execution and data acquisition. A.S. Piotrowski‐Daspit and C.A. Aguilar provided experimental assistance and resources. M.L. Killian contributed intellectual input. **C. Loebel** supervised the project, provided conceptual guidance, secured funding, and critically revised the manuscript. M.L. Killian and C. Loebel jointly contributed to funding acquisition. All authors contributed to the review and editing of the manuscript and approved the final version for submission.

## Conflicts of Interest

The authors declare no conflicts of interest.

## Supporting information




**Supporting File 1**: advs77895‐sup‐0001‐SuppMat.docx.

**Supporting File 2**: advs77895‐sup‐0002‐SuppMat.xlsx.

**Supporting File 3**: advs77895‐sup‐0003‐SuppMat.xlsx.

## Data Availability

The proteomics data generated in this study have been deposited in the Dryad Digital Repository under https://doi.org/10.5061/dryad.0k6djhbfj. Additional data that support the findings of this study are available from the corresponding author upon reasonable request.
