# Supplementary material for: Nascent Extracellular Matrix Converts Biomaterial Cues into Cell Fate Decisions
Source: Adv Sci (Weinh). 2026 Sep 27:e77895. Online ahead of print. doi: 10.1002/advs.77895 (PMC13616248; doi:10.1002/advs.77895)
Supplement: Supplementary file 1 [file ADVS-9999-e77895-s002.docx]

**Nascent extracellular matrix converts biomaterial cues into cell fate decisions**

J.Y. Liu^1,2^, Ziqian Yang^1,2^, Arina Velieva^1,2^, E. M. Plaster^3^, M. Fan^1,2^, D. W. Ahmed^3^, A. Roy^2,4^, P. Duran^3^, P. Panovich^3^, A. S. Piotrowski-Daspit^3,5^, C. A. Aguilar^3^, M. L. Killian^6^, and C. Loebel^1,2,4^*

^1^Department of Bioengineering, University of Pennsylvania, USA

^2^Center for Precision Engineering for Health (CPE4H), University of Pennsylvania, USA

^3^Department of Biomedical Engineering, University of Michigan, USA

^4^Department of Materials Science & Engineering, University of Michigan, USA

^5^Internal Medicine - Pulmonary & Critical Care Medicine Division, University of Michigan, USA

^6^Department of Orthopaedic Surgery, University of Michigan, USA

^*^Corresponding author: loebelcl@engineering.upenn.edu

**Figure S1. nECM deposition heterogeneity over the culture period.** **a-c.** Histograms of nECM thickness distribution of chondrocytes cultured in low, mid and high modification hydrogels at day 1, 4, 7 (bin width = 0.4 μm. Red arrows indicate percentages of cells with non-detectable (ND) nECM, Day 1: low *n* = 85, *N* = 2; mid *n* = 86, *N* = 2; high *n* = 89, *N* = 2, Day 4: low *n* = 82, *N* = 2; mid *n* = 86, *N* = 2; high *n* = 102, *N* = 2, Day 7: low *n* = 101, *N* = 3; mid *n* = 136, *N* = 3; high *n* = 122, *N* = 3). **a-c.** *N* = number of independent experiments

**Figure S2. YAP nuclear-to-cytoplasmic (N/C) ratio.** Quantification of YAP nuclear-to-cytoplasmic (N/C) ratio in chondrocytes encapsulated in low and high hydrogels at day 7.low: n = 29 cells, *N* = 1; and high: n = 26 cells, *N* = 1) *N* = number of independent experiments, Box plots = median, interquartile range, and minimum–maximum values, ns: no significant difference by Student’s *t-test*.

**Figure S3. SOX9 gene expression.** SOX9 gene expression measured by qPCR at day 7, normalized to S18 housekeeping gene using the ΔΔCt method. Data are expressed as fold change relative to low modification hydrogels (set as 1.0). (*N* = 3). *N* = number of independent experiments, error bar = standard deviation, ****p < 0.0001 by Student’s *t-test*.

**Figure S4. Hydrogel physical properties of ‘low’ and ‘high’ 5kPa NorHA hydrogel. a.** Stress relaxation curves of ‘low’ and ‘high’ hydrogels under 15% compressive strain over 1000 s. Data were normalized to the initial stress at *t* = 0 s (N = 4). **b.** Quantification of HiLyte 488-conjugated fibronectin adsorption after 48 h. Fluorescence intensity was normalized to the serum-free medium-only background control (N = 3 independent experiments, n = 15 ROIs). **c.** Swelling ratio of ‘low’ and ‘high’ hydrogels incubated at 37°C over 3 days (N = 5). **d.** Representative fluorescence recovery after photobleaching (FRAP) images of FITC-dextran diffusion following hydrogel swelling and quantification of diffusivity (n = 5). *N* = number of independent experiments. **b, d:** Box plots = median, interquartile range, and minimum–maximum values, ns: no significant difference by Student’s *t*-test. **c:** error bar = standard deviation.

**Figure S5. nECM deposition and Sox9 nuclear localization in 10 kPa hydrogels. a.** Representative fluorescent images and quantification of nECM area in the cross-section of chondrocytes cultured in low- and high-modification 10 kPa hydrogels at day 7 (scale bar: 10 μm, low: n = 73 cells, N = 2; and high: n = 66 cells, N = 2). **b.** Representative immunofluorescent images and quantification of Sox9 nuclear translocation calculated by the nucleus-to-cytoplasm (NC) ratio at day 7 (scale bar: 10 μm, low: n = 86 cells, N = 2; and high: n = 87 cells, N = 2). **a–b.** *N* = number of independent experiments, box plots = median, interquartile range, and minimum–maximum values, ****p < 0.0001 by two-tailed Mann-Whitney test.

**Figure S6. Chondrogenic gene expression and cell morphology following CD44 inhibition. a.** Ratio of COL2A1/COL1A1 and ACAN/VCAN gene expression at day 7, measured by qPCR (N = 3). b. Quantification of chondrocyte cell aspect ratio at day 7 (low-Ctrl: n = 80 cells, N = 2; and low-CD44i: n = 82 cells, N = 2). **a-b.** *N* = number of independent experiments, error bars = standard deviation. a: *p < 0.05, ns: not significant by one-way ANOVA with Tukey's multiple comparisons test. b: ns: no significant difference by two-tailed Mann-Whitney test. Box plots = median, interquartile range, and minimum–maximum values.

**Figure S7. CD44 blocking in ‘high’ hydrogels.**  **a.** Representative fluorescent images and quantification of the incorporation of EdU in chondrocytes cultured without (Ctrl) and with CD44 inhibition (CD44i) in high modification hydrogels at day 7. (scale bar = 100μm, high-Ctrl: *n* = 23 ROIs, *N* = 3; high-CD44: *n* = 25 ROIs, *N* = 2) **b.** Representative fluorescent images (dashed line outlines nuclei) and quantification of Sox9 NC ratio of chondrocytes cultured without (Ctrl) and with CD44 inhibition (CD44i) in low modification hydrogels at day 7. (scale bar = 10μm, high-Ctrl: *n* =124 cells, *N* = 4; high-CD44: *n* = 117 cells, *N* = 4). **a-b.** *N* = number of independent experiments, ns: no significant difference by **a**. Two-tailed Student’s *t*-test; **b.** two-tailed Mann–Whitney test. Box plots show median, interquartile range, and minimum–maximum values.

**Figure S8. UMAP analysis of spatial protein features.** **a.** UMAP projection of quantified spatial features from aggrecan (ACAN), collagen type II (COL2), collagen type VI (COL6), and decorin (DCN) in low hydrogels. **b.** UMAP projection of the same spatial features in high hydrogels.

**Figure S9. Additional spatial quantifications of chondrogenic proteins.** **a.** Quantification of protein extension for collagen type VI (low: n = 57 cells; high: n = 36 cells), decorin (low: n = 43 cells; high: n = 30 cells), and aggrecan core protein (low: n = 35 cells; high: n = 36 cells). **b.** Quantification of protein area for collagen type II (low: n = 37 cells; high: n = 29 cells), collagen type VI (low: n = 57 cells; high: n = 36 cells), and decorin (low: n = 31 cells; high: n = 30 cells). **c.** Quantification of pericellular coverage for collagen type II (low: n = 37 cells; high: n = 29 cells), decorin (low: n = 43 cells; high: n = 30 cells), and aggrecan core protein (low: n = 32 cells; high: n = 32 cells). **d.** Quantification of the pericellular-to-distal integrated signal ratio for collagen type II (low: n = 38 cells; high: n = 26 cells), collagen type VI (low: n = 58 cells; high: n = 35 cells), and aggrecan core protein (low: n = 34 cells; high: n = 38 cells). **a–d.** ****p < 0.0001, ***p < 0.001, **p < 0.01, ns: not significant by two-tailed Mann–Whitney test. Box plots = median, interquartile range, and minimum–maximum values.

**Figure S10. Isotype controls and nECM deposition following integrin β1 inhibition.** **a.** Quantification of the Sox9 nuclear-to-cytoplasmic (N/C) ratio in chondrocytes cultured in ‘low’ hydrogels under untreated control (Ctrl), mouse IgG isotype control (MIgG), or rat IgG isotype control (RIgG) conditions at day 7 (Ctrl: n = 81 cells, N = 2; MIgG: n = 83 cells, N = 2; RIgG: n = 91 cells, N = 2). **b.** Quantification of nECM thickness in chondrocytes cultured in ‘low’ and ‘high’ hydrogels with or without integrin β1 inhibition (ITGB1i) at day 7. Data were normalized to the mean nECM thickness of the ‘low’ Ctrl group (low-Ctrl: n = 123 cells, N = 4; low-ITGB1i: n = 75 cells, N = 2; high-Ctrl: n = 150 cells, N = 4; high-ITGB1i: n = 72 cells, N = 2). N = number of independent experiments. **a-b.** ****p < 0.0001, *p < 0.05, ns: not significant by Kruskal–Wallis test with Dunn's multiple-comparisons test. Box plots show the median, interquartile range, and minimum–maximum values.

**Figure S11. Combined CD44 and integrin β1 inhibition on Sox9 nuclear localization.** **a.** Quantification of the Sox9 nuclear-to-cytoplasmic (N/C) ratio in chondrocytes cultured in **high** hydrogels under untreated control (Ctrl), CD44 inhibition (CD44i), integrin β1 inhibition (ITGB1i), or combined CD44 and integrin β1 inhibition (ITGB1i + CD44i) at day 7 (Ctrl: n = 124 cells, N = 4; CD44i: n = 117 cells, N = 4; ITGB1i: n = 138 cells, N = 4; ITGB1i + CD44i: n = 138 cells, N = 3). **b.** Quantification of the Sox9 nuclear-to-cytoplasmic (N/C) ratio in chondrocytes cultured in **low** hydrogels under untreated control (Ctrl), CD44 inhibition (CD44i), integrin β1 inhibition (ITGB1i), or combined CD44 and integrin β1 inhibition (ITGB1i + CD44i) at day 7 (Ctrl: n = 138 cells, N = 4; CD44i: n = 110 cells, N = 4; ITGB1i: n = 133 cells, N = 4; ITGB1i + CD44i: n = 91 cells, N = 3). N = number of independent experiments. **a-b.** ****p < 0.0001, ***p < 0.001, **p < 0.01, *p < 0.05, ns: not significant by Kruskal–Wallis test with Dunn's multiple-comparisons test. Box plots = median, interquartile range, and minimum–maximum values.

**Figure S12. Chemical characterization of norbornene-modified hyaluronic acid (NorHA) polymer. a.** Representative ^1^H NMR spectrum of NorHA with ‘low’, ‘mid’, and ‘high’ modification. Orange-shaded regions indicate sugar ring protons of HA, while grey-shaded regions correspond to vinyl proton peaks of norbornene groups^28^. **b.** Quantification of the degree of substitution (DS) for the NorHA batches used in this study (‘low’: *n* = 3, *N* = 3; mid’: *n* = 6, *N* = 6, ‘high’: *n* = 5, *N* = 5). *N* = number of independent experiments, error bar = standard deviation, **b:** ****p < 0.0001 by one-way ANOVA with Tukey’s multiple comparisons test.

**Figure S13. Characterization of norbornene-modified alginate (NorAlg) polymer and hydrogel. a.** ^1^H NMR spectra of Low- and High-modified NorAlg. The cyan-shaded region indicates the formyl proton peak of the dimethylformamide (DMF) internal standard and the grey-shaded regions correspond to the vinyl proton peaks of the norbornene groups. **b.** Quantification of storage moduli of Low- and High-modified NorAlg (*N* = 3 hydrogels). error bar = standard deviation, ns: not significant by Student’s *t-test*.

**Table S1. Proteomic dataset of chondrocytes cultured in ‘low’ and ‘high’ hydrogels.**
Proteomic data were collected at day 7 and include all identified proteins and the subset classified as matrisome proteins. Abundance values for all identified proteins are provided for three biological replicates per condition. Matrisome proteins are annotated by division and category, with normalized abundance, z-score, fold change, log2 fold change, *p*-value, and adjusted *p*-value.

**Table S2. Features used for UMAP analysis of spatial nascent ECM organization.**
Definitions of quantitative image-derived features used for UMAP analysis of single-cell nascent ECM organization.
